# The effect of melatonin supplementation on glycemic control in patients with type 2 diabetes

**DOI:** 10.3389/fendo.2025.1572613

**Published:** 2025-07-08

**Authors:** Xutong Lv, Huiqi Sun, Shuang Ai, Dongbin Zhang, Hongxiu Lu

**Affiliations:** ^1^ Master Degree Candidate, First School of Clinical Medicine, Shandong University of Traditional Chinese Medicine, Jinan, China; ^2^ Department of Anesthesiology, Shandong University of Traditional Chinese Medicine Affiliated Hospital, Jinan, China

**Keywords:** melatonin, fasting plasma glucose, glycosylated hemoglobin, type 2 diabetes mellitus, T2DM

## Abstract

**Background and purpose:**

Melatonin supplementation has shown potential benefits in the management of diabetes in clinical trials; however, prior meta-analyses have not specifically focused on individuals with type 2 diabetes mellitus (T2DM). This study investigates the efficacy of melatonin supplementation in improving glycemic control among patients with T2DM by systematically reviewing and analyzing data from randomized controlled trials (RCTs).

**Methods:**

A comprehensive literature search was conducted in PubMed, Cochrane Library, Scopus, Web of Science, and Embase from their inception to September 2024. RCTs evaluating the effects of melatonin supplementation in adults diagnosed with T2DM were included. The methodological quality of the studies was assessed using the Cochrane Risk of Bias Tool. Data were synthesized and analyzed using RevMan version 5.3.

**Results:**

A total of nine RCTs were included in the meta-analysis (n=9). These studies collectively involved 427 participants. Melatonin supplementation was associated with a statistically significant reduction in glycated hemoglobin (HbA1c) levels compared to placebo [mean difference [MD]: -0.65; 95% CI: -1.28, -0.02; P = 0.04], However, no significant effect was observed on fasting plasma glucose (FPG) levels [mean difference: -6.40; 95% CI: -15.79, 2.99; P = 0.18].

**Conclusion:**

This meta-analysis suggests that melatonin supplementation significantly reduces HbA1c levels in patients with type 2 diabetes mellitus compared to placebo, indicating potential benefits for long-term glycemic control. However, no significant effect was observed on FPG levels.

**Systematic review registration:**

https://www.crd.york.ac.uk/PROSPERO/, identifier CRD42024629557.

## 1 Introduction

Type 2 diabetes mellitus (T2DM) is a chronic metabolic disorder characterized by insulin resistance and progressive β-cell dysfunction, leading to sustained hyperglycemia and multiple systemic complications. Over the past decades, the global prevalence of T2DM has risen sharply, reflecting both demographic transitions and lifestyle changes. In 2000, diabetes affected approximately 2.8% of the global population; by 2030, this figure is projected to increase to 4.4% (1). As of 2017, diabetes ranked as the ninth leading cause of mortality, with an incidence of 6,059 cases per 100,000 population and more than one million diabetes-related deaths reported annually (2). Notably, the burden of T2DM is shifting toward developing countries, where over 75% of individuals with diabetes are expected to reside by 2025 (3).

The clinical burden of T2DM extends far beyond hyperglycemia. Long-term complications such as cardiovascular disease, nephropathy, retinopathy, and neuropathy significantly compromise quality of life and increase the risk of disability and premature death (4). Individuals with T2DM face a threefold higher risk of developing cardiovascular disease compared to those without diabetes (5). While pharmacological treatments—particularly oral hypoglycemic agents—remain the mainstay of T2DM management, their use is often associated with reduced efficacy over time, adverse effects (e.g., gastrointestinal discomfort), and, in some cases, toxicity (6). These limitations have prompted growing interest in adjunctive or alternative therapies aimed at improving glycemic control and mitigating complications with fewer side effects.

Melatonin, an indoleamine primarily secreted by the pineal gland, plays a central role in the regulation of circadian rhythms and the sleep-wake cycle, and is widely recognized for its potent antioxidant and anti-inflammatory properties (7). Beyond its chronobiological functions, melatonin contributes to cellular protection by scavenging free radicals, attenuating oxidative stress, and modulating the activity of antioxidant enzymes (8, 9). Recent research has expanded the potential therapeutic scope of melatonin, suggesting its involvement in glucose metabolism and insulin regulation. Mechanistic studies indicate that melatonin may influence the pathophysiology of T2DM via multiple intracellular signaling pathways, including cyclic adenosine monophosphate (cAMP), cyclic guanosine monophosphate (cGMP), inositol trisphosphate (IP3), and transcription factor-regulated cascades (10). These findings have prompted interest in melatonin as a possible adjunctive intervention in T2DM management. However, the role of melatonin in glucose homeostasis remains controversial. Some evidence suggests that elevated melatonin levels may impair insulin secretion, particularly among individuals with specific genetic polymorphisms. For example, a study from Lund University demonstrated that increased melatonin concentrations were associated with reduced insulin secretion, with this effect being more pronounced in carriers of certain MTNR1B gene variants (11). Although previous meta-analyses (12, 13) have examined the association between melatonin supplementation and metabolic parameters in diabetic populations, these studies present two notable limitations. First, they did not differentiate between type 1 diabetes mellitus and T2DM, instead pooling data from both conditions. This methodological approach overlooks critical differences in pathophysiological mechanisms and melatonin responsiveness between the two disease types (14). For instance, insulin resistance—characteristic of T2DM—may alter melatonin receptor signaling pathways, potentially leading to differential therapeutic outcomes (15). Second, several randomized controlled trials (RCTs) published in recent years have specifically investigated the effects of melatonin supplementation in individuals with T2DM. However, these studies have not yet been comprehensively synthesized in a dedicated meta-analysis. Given the emergence of these new data and the need to clarify melatonin’s role in the context of T2DM specifically, an updated and focused meta-analysis is warranted to provide a more accurate and clinically relevant evaluation.

This study systematically identified, reviewed, and synthesized RCTs assessing the efficacy of melatonin supplementation in the management of T2DM. Through a comprehensive meta-analysis, it aims to provide an updated and evidence-based evaluation of melatonin’s therapeutic potential, thereby informing clinical decision-making and guiding future research directions.

## 2 Information and methods

### 2.1 Literature search methods

This study was conducted in accordance with the PRISMA statement (16) and evaluated for the AMSTAR guidelines (17). The protocol was registered in the PROSPERO under registration number CRD42024629557. A comprehensive literature search was performed across five major electronic databases: PubMed, Cochrane Library, Scopus, Web of Science, and Embase, covering all available records from database inception to September 2024. The search strategy incorporated both Medical Subject Headings (MeSH) and free-text terms, and was structured using Boolean operators (“AND”, “OR”) to maximize sensitivity. The core search terms included the MeSH descriptors “Type 2 Diabetes Mellitus” and “Melatonin”. These were supplemented with relevant synonyms and variations, such as: “Diabetes Mellitus, Adult-Onset”, “Adult-Onset Diabetes Mellitus”, “Stable Diabetes Mellitus”, “Diabetes Mellitus, Type II”, “Non-Insulin-Dependent Diabetes Mellitus (NIDDM)” etc. To identify RCTs, the search was further refined using terms including “Randomized Controlled Trial”, “Randomized” and “Placebo”. No restrictions were imposed regarding language or publication status. The complete search strategy for each database is available in **Supplementary Material** to ensure transparency and reproducibility.

### 2.2 Inclusion and exclusion criteria

Inclusion Criteria: (1)This study examined the effects of melatonin supplementation on patients with T2DM using a RCT design, published in any language; (2) The study involved patients with T2DM of any gender, age, or nationality; (3) The intervention involved melatonin supplementation, either alone or in combination with other agents; (4) The primary outcomes included glycemic parameters, such as glycated hemoglobin (HbA1c), fasting plasma glucose (FPG), and insulin resistance.

Exclusion criteria: (1) Studies involving patients without T2DM or those receiving other interventions unrelated to melatonin supplementation; (2) Research for which there was no complete text available or where the outcome measures were not complete; (3) Studies that were not RCTs, including systematic reviews, mechanistic studies, conference abstracts, and animal studies.

### 2.3 Literature screening and data extraction

Two independent reviewers conducted the initial screening of retrieved records by evaluating titles and abstracts against the predefined inclusion and exclusion criteria. Full-text articles were subsequently assessed to determine final eligibility. Any discrepancies between the two reviewers were resolved through discussion, and if necessary, by consulting a third reviewer to reach consensus. EndNote X9 software was employed for reference management. Data extraction was carried out using a standardized Microsoft Excel spreadsheet, which was designed to collate retrieved studies, remove duplicates and screen records. This process was performed independently by both reviewers to ensure accuracy and consistency.

### 2.4 Quality evaluation:

The methodological quality of the included RCTs was assessed using Review Manager (RevMan) version 5.3, following the guidelines outlined in the Cochrane Handbook for Systematic Reviews of Interventions (version 5.1).

### 2.5 Statistical analysis:

For all included studies, continuous outcomes such as HbA1c and FPG were reported as mean( $\bar{\text{x}}$ ) ± standard deviation (SD) values before and after the intervention. When outcomes were presented as standard errors of the mean (SEM), SDs were derived using the formula: SD = SEM × √n, where *n* denotes the sample size of the corresponding group. Effect sizes for continuous variables were expressed as mean differences (MDs) with 95% confidence intervals (CIs). Inter-study heterogeneity was assessed using Cochrane’s Q test and the I² statistic (18, 19). A fixed-effects model was applied when heterogeneity was low (*I² ≤ 50%* and *P > 0.10*), whereas a random-effects model was used in cases of substantial heterogeneity (*I² > 50%* and/or *P < 0.10*). Where applicable, FPG values reported in mmol/L were converted to mg/dL for consistency. Publication bias was evaluated visually through funnel plots and statistically using Egger’s test, conducted in Stata version 18.0. A *P* > 0.05 was considered indicative of no significant publication bias. Sensitivity analyses were also conducted in Stata to assess the robustness of the pooled results.For studies that did not report post-intervention SDs, missing values were estimated using an imputed correlation coefficient (*r = 0.5*) between baseline and follow-up measures, in accordance with Cochrane Handbook recommendations. Studies for which variability data could not be obtained or reliably estimated—and where authors could not be contacted—were excluded from quantitative synthesis and included only in narrative analysis.

## 3 Results

### 3.1 Literature search

**Figure 1:**
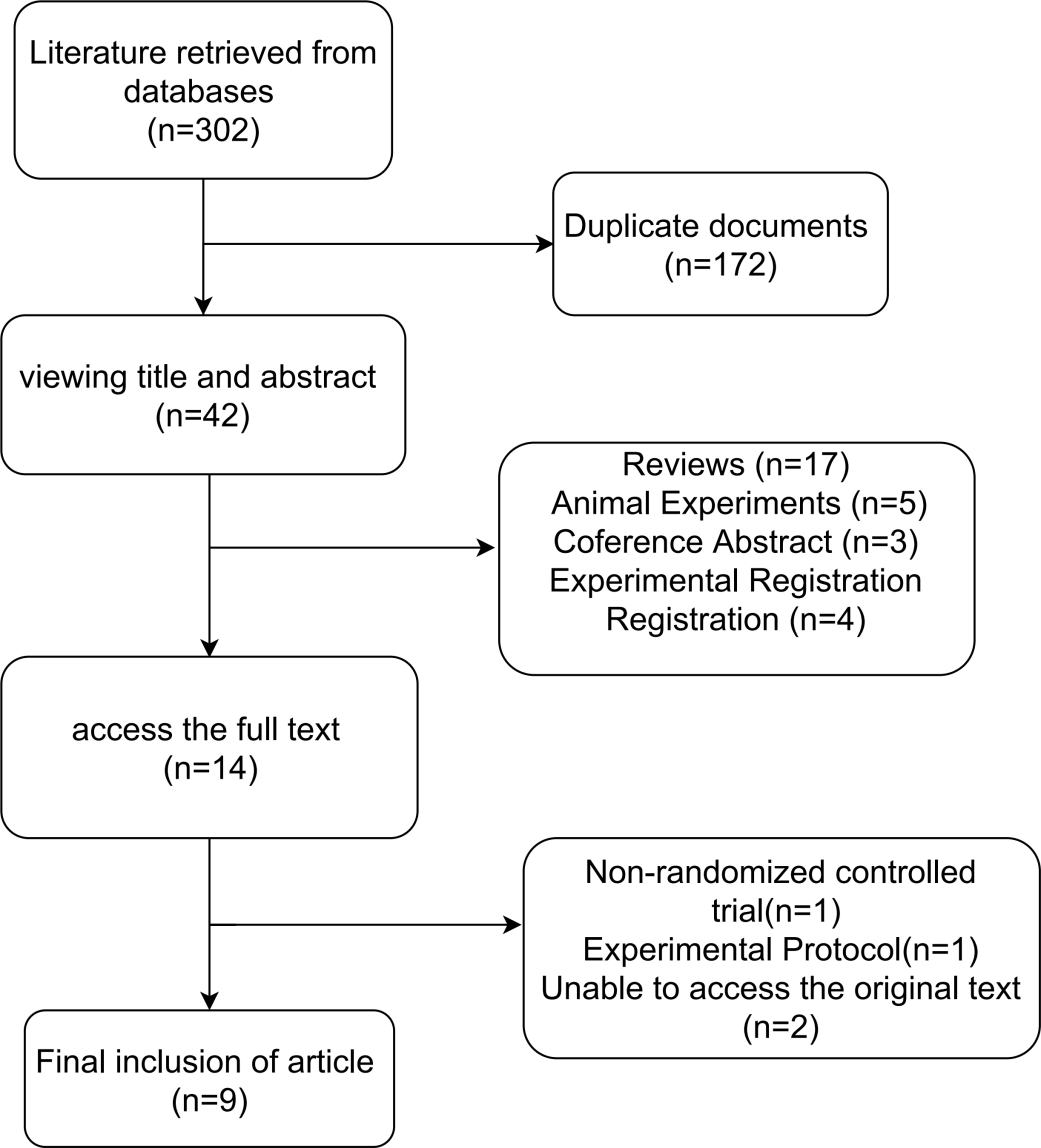
Flowchart of the screening of literature.

A total of 302 records were initially retrieved through database searches. After removing duplicates, 130 unique articles remained. Following title and abstract screening, 42 articles were excluded for failing to meet the predefined inclusion criteria. Subsequently, full-text review led to the exclusion of one article due to ineligible study design, and two additional studies were excluded due to inaccessible full texts. Ultimately, nine RCTs met all eligibility criteria and were included in the meta-analysis(n=9), comprising a total of 427 participants diagnosed with T2DM. The detailed study selection process is depicted (**Figure 1**). The baseline characteristics, intervention details, and primary outcomes of the included studies are summarized (**Table 1**).

**Table 1:** Detailed description of the basic characteristics and main results of 9 RCTS included.

First author/year	Study design	Sample	Age	Gender (M/F)	Intervention duration	Intervention	Main results
Diana-Maria Anton (20) (2021)	Randomized Double- blind	54 patients with T2DM with symptoms of periodontal disease	T:53.24 ± 3.4 C:52.21 ± 3.1	T:60%/40%C:56%/44%	8 weeks	T:melatonin(6mg) C:placebo(6mg)	①
Bazyar, Hadi (21) (2022)	Randomized Double- blind	50 patients with type 2 diabetes with symptoms of periodontal disease	T:53.72 ± 6.68 C:51.45 ± 0.03	T:46.75%/53.3% C:53.3%/46.7%	8 weeks	T:melatonin(6mg) C:placebo(6mg)	①
Amir Farrokhian (22) (2019)	Randomized Triple-blind	70 patients with type 2 diabetes	T:57.74 ± 8.57 C:57.61 ± 9.11	T:17/17 C:20/16	8 weeks	T:melatonin(6mg) C:placebo(6mg)	①②
Doron Garfinkel (23) (2011)	Randomized Double- blind Crossover	36 patients with type 2 diabetes mellitus with insomnia	63 ± 8	11/25	7weeks+20weeks	melatonin(2mg) - elution - placebo(2mg)/placebo(2mg)- elution -melatonin(2mg) melatonin(2mg)	①
Saad A Hussain (24) (2006)	Randomized Double- blind	46 patients with type 2 diabetes	49.1 ± 6.0	A: 8/7 B: 11/7 C: 7/6	12 weeks	A:placebo + metformin B:melatonin(10mg) + zinc(50mg) + metformin C:melatonin(10mg) + zinc(50mg)	①②
Esben S. Lauritzen (25) (2022)	Randomized Double- blind Crossover	17 patients with type 2 diabetes	41-70	male patients	12 weeks	melatonin(10mg) - elution - placebo(10mg)	③
Wagner Martorina (26) (2023)	Randomized Double- blind Crossover	30 patients with type 2 diabetes	A:62,27 ± 8,20 B:61,87 ± 10,95	A:6/9 B:8/7	3 weeks	A:melatonin(3mg) - elution - placebo(3mg) B:placebo(3mg)- elution -melatonin(3mg)	④
Fariba Raygan (27) (2019)	Randomized Double- blind	60 patients with type 2 diabetes mellitus coronary heart disease	T:67.7 ± 11.4 C:65.3 ± 10.1	T:16/14 C:17/13	12 weeks	T:melatonin(10mg) C:placebo(10mg)	②⑤
Mohammad Reza Rezvanfar (28) (2016)	Randomized Double- blind	64 patients with type 2 diabetes	52 ± 8	23.5%/76.5%	24 weeks	placebo(6mg)- elution -melatonin(6mg)	①②

① glycated hemoglobin (HbA1c) ② fasting plasma glucose (FPG) ③ insulin sensitivity ④ glycemic Variability ⑤ quantitative insulin sensitivity check index.

### 3.2 Main findings

Among the ten included studies, six demonstrated statistically significant improvements in diabetes-related metabolic parameters following melatonin supplementation. Specifically, six trials reported reductions in HbA1c levels, while three trials observed decreases in FPG. Conversely, one study that assessed insulin sensitivity as the primary outcome reported a decline in insulin sensitivity after melatonin administration. Additionally, a separate trial evaluating glycemic variability as the main endpoint found an increase in glycemic fluctuations associated with melatonin use. Notably, one study documented a significant enhancement in insulin sensitivity as measured by the Quantitative Insulin Sensitivity Check Index.

### 3.3 Meta-analysis

**Figure 2:**
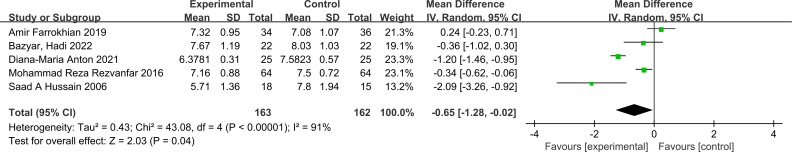
Forest plots of melatonin supplementation’s effects on HbA1C.

**Figure 3:**
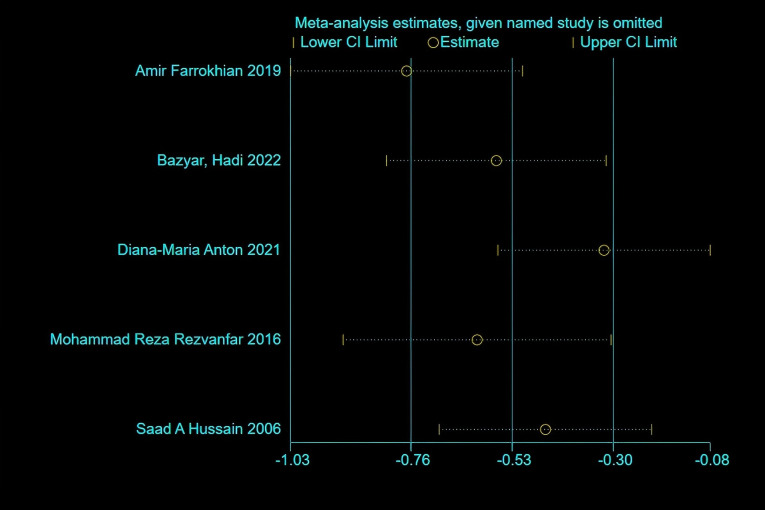
Sensitivity analysis of HbA1c.

HbA1c: Five studies reported changes in HbA1c levels, encompassing 163 participants in the melatonin intervention groups and 162 in the control groups. Significant heterogeneity was detected among studies (P < 0.00001, I² = 91%), warranting the use of a random-effects model for meta-analysis. The pooled estimate indicated melatonin supplementation showed a beneficial effect on HbA1c compared to placebo [MD: -0.65; 95% CI: -1.28, -0.02; P=0.04](**Figure 2**). However, given the proximity of the confidence interval upper limit to the null value and the borderline statistical significance, these findings should be interpreted with caution. Sensitivity analysis, conducted via a leave-one-out approach, demonstrated consistent directionality of effect with all pooled MDs remaining negative and 95% CIs not crossing zero—the highest upper bound reaching only -0.08 (**Figure 3**). Notably, the exclusion of the study by Diana-Maria Anton (2021) resulted in a confidence interval with an upper limit closest to zero, indicating this study had the greatest influence on the pooled estimate. However, its removal did not reverse the direction of the effect. The exclusion of other individual studies yielded minimal changes, implying limited influence on the overall meta-analytic estimate.

**Figure 4:**
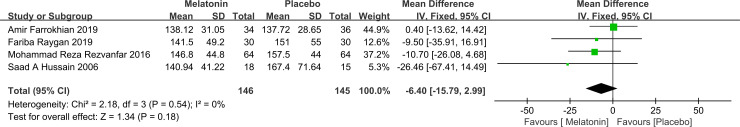
Forest plots of melatonin supplementation’s effects on FPG.

FPG: Four studies reported changes in FPG, including a total of 146 participants in the intervention group and 145 in the control group. Heterogeneity analysis indicated no significant between-study variability (P = 0.54; I² = 0%), justifying the use of a fixed-effects model. The pooled analysis showed that melatonin supplementation did not lead to a statistically significant reduction in FPG levels compared to placebo [MD = -6.40; 95% CI: -15.79, 2.99; P = 0.18] (**Figure 4**). It is important to note that the limited sample size may reduce statistical power, increasing the risk of Type II error and potentially obscuring a true effect.

### 3.4 Quality assessment of the included studies

The risk of bias across the included studies was evaluated using the Cochrane Risk of Bias Assessment Tool. Among the studies, six explicitly reported the use of random allocation, while three mentioned randomization without specifying the method for sequence generation. Additionally, three studies did not provide sufficient information regarding allocation concealment. All included studies reported the implementation of blinding. No evidence of reporting bias was identified.

**Figure 5:**
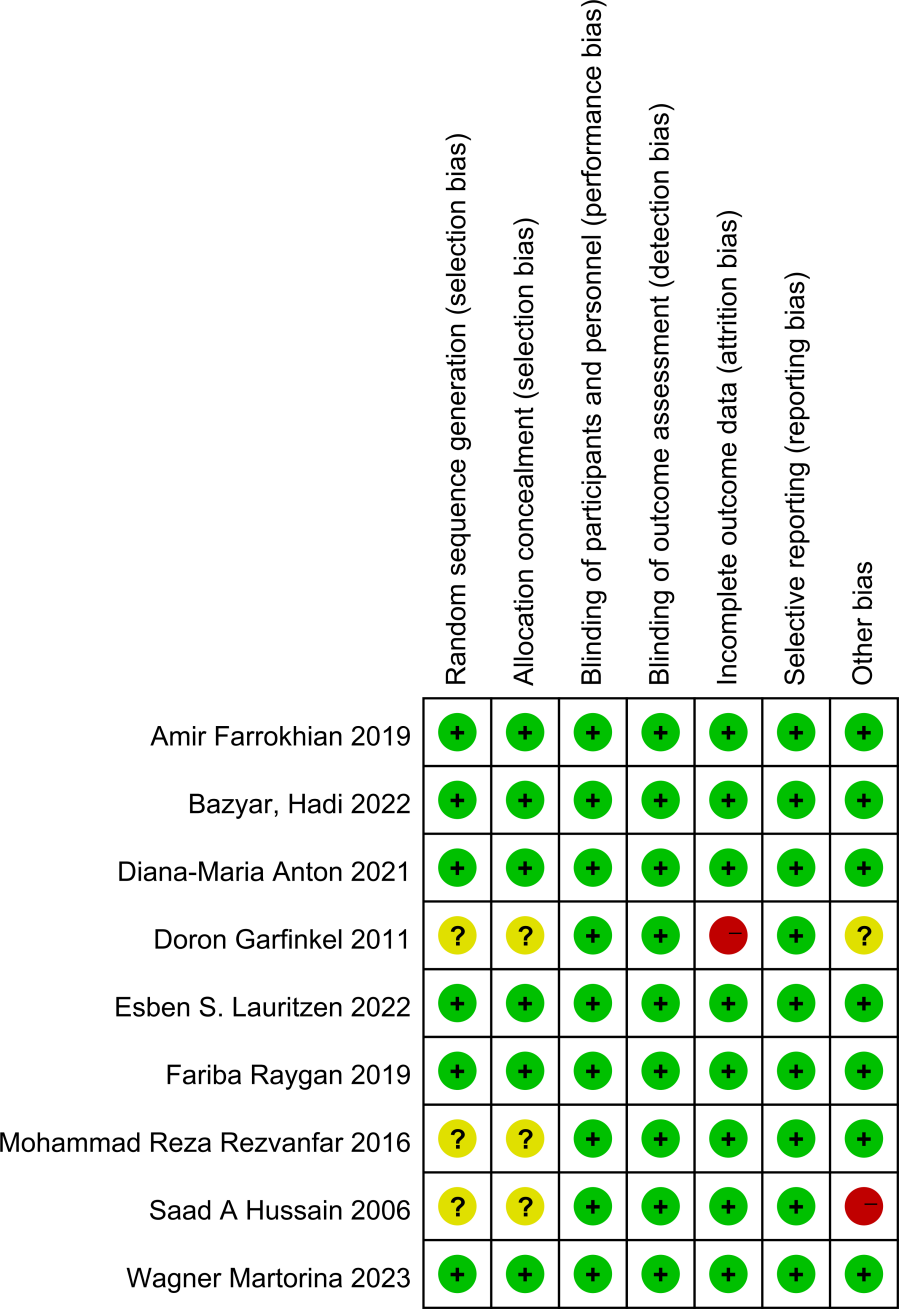
Summary of the risk of bias in the included studies.

**Figure 6:**
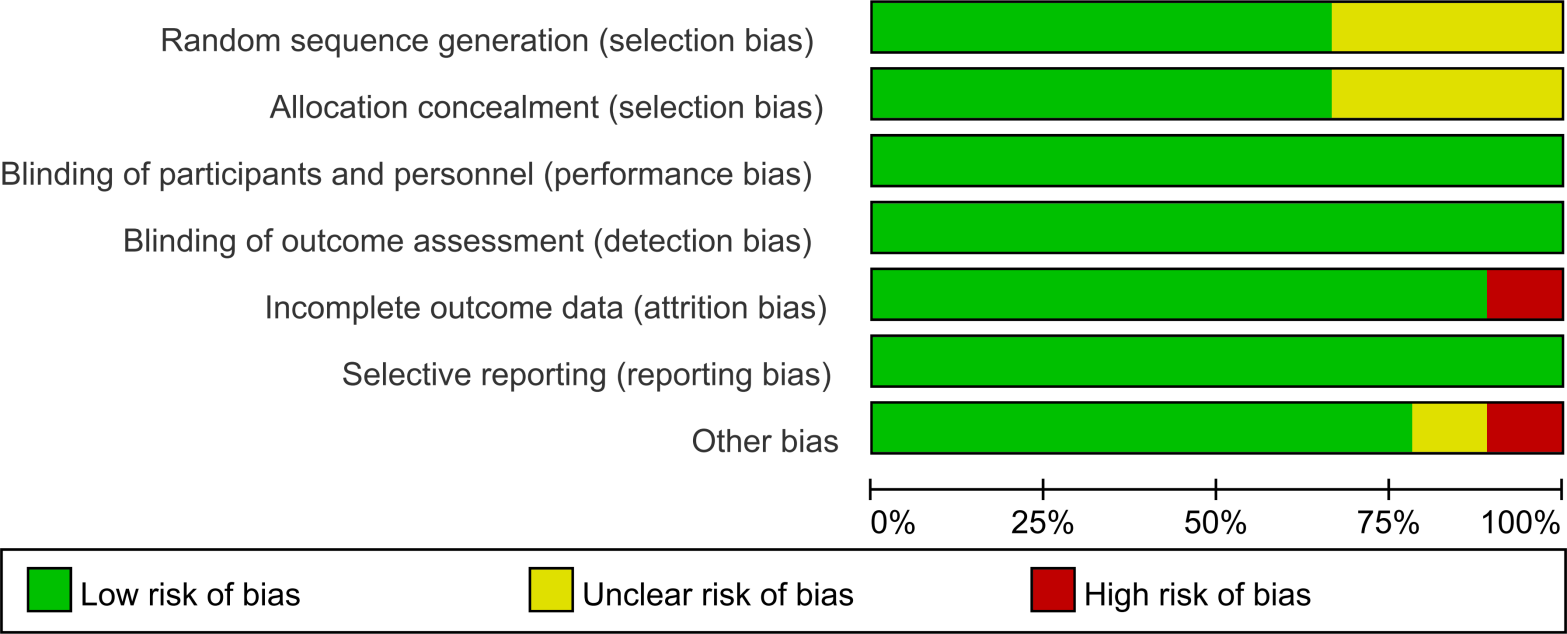
Graph of the risk of bias in the included studies.

**Figure 7:**
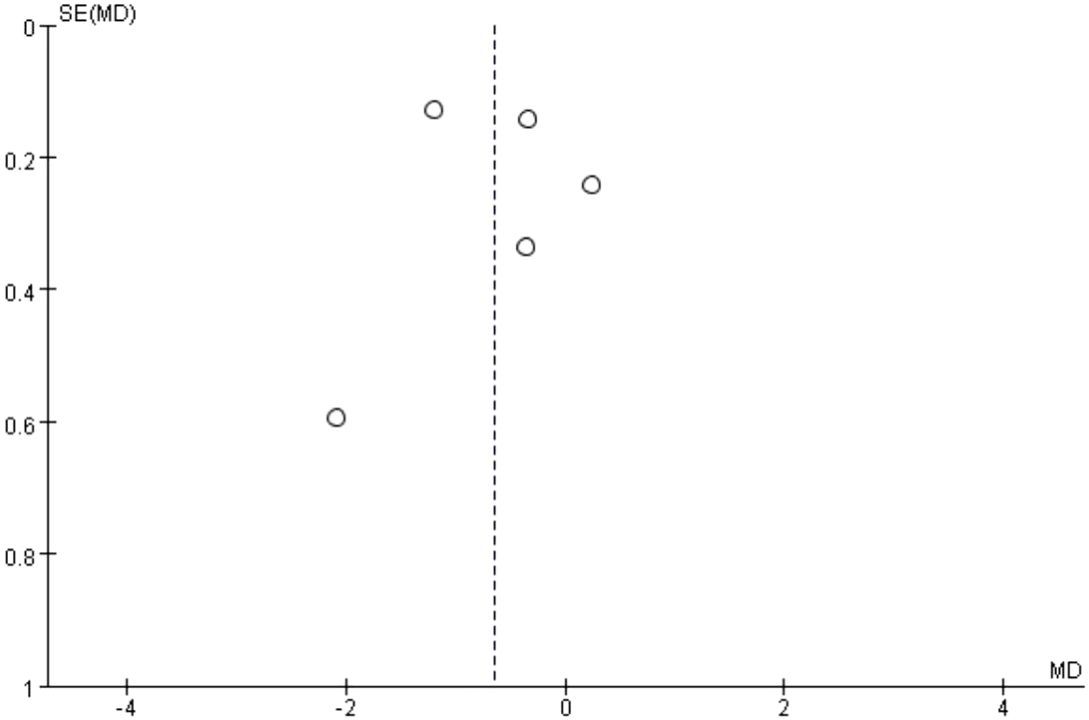
HbA1c publication bias funnel plot.

The results of the risk of bias assessment are summarized in **Figures 5** and **6**, which present the evaluations according to the Cochrane tool criteria (**Figures 5**, **6**). Publication bias for the outcome of HbA1c was assessed using a funnel plot (**Figure 7**), and the Egger’s test yielded a P-value of 0.236, indicating no statistically significant evidence of publication bias.

## 4 Discussion

This study is the first to systematically restrict inclusion to RCTs conducted exclusively in individuals with T2DM, a focused approach that holds significant clinical relevance. Mechanistically, T2DM is primarily characterized by impaired insulin secretion. Melatonin has been shown to improve pancreatic β-cell function by modulating the expression of the MTNR1B gene, which plays a particularly critical role in the pathophysiology of T2DM (29). From a clinical standpoint, T2DM accounts for more than 90% of all diabetes cases, highlighting the need for population-specific evidence to guide the use of melatonin as a therapeutic supplement. This type-specific analysis compensates for the limitations of previous studies that mixed different types of diabetes.

Our meta-analysis demonstrates that melatonin supplementation significantly improves HbA1c levels in patients with T2DM, while no statistically significant effect was observed on FPG. This finding contrasts with the results of previous meta-analyses (12) involving mixed diabetic populations. The discrepancy may be attributed to the absence of diabetes type-specific subgroup analyses in earlier studies, leading to potential confounding effects from type 1 diabetes mellitus data. Furthermore, our analysis identified potential adverse effects of melatonin in T2DM patients. For instance, Lauritzen et al (25) reported a reduction in insulin sensitivity following melatonin administration, while Martorina et al (26) observed increased glycemic variability. Given the limited number of studies included in the present analysis, further high-quality RCTs are warranted to validate these findings and explore underlying mechanisms.

Although the analysis of HbA1c yielded statistically significant results, the upper bound of the confidence interval approached the line of no effect, indicating that the statistical significance was relatively modest. This study also conducted a risk of bias assessment of the included trials. Notably, the meta-analysis of HbA1c exhibited substantial heterogeneity (I² = 91%), suggesting considerable variability across studies. Potential sources of heterogeneity may include differences in melatonin dosage, duration of intervention, baseline characteristics of participants, and methodological quality of the studies. Some studies have certain risks of bias in aspects such as blind execution, hidden allocation, and the completeness of result reporting, which may affect the accuracy of effect size estimation. Therefore, while the pooled results indicate that melatonin may exert a beneficial effect on HbA1c, this conclusion should be interpreted with caution, taking into account both the risk of bias and the methodological rigor of the included studies to avoid overstatement of the findings.

Preclinical evidence supports the potential of melatonin in modulating glycemic control. An animal study (30) demonstrated that melatonin treatment significantly improved hyperglycemia in T2DM rat models by reducing insulin levels, inhibiting hemoglobin glycation, and ameliorating insulin resistance. These effects may be attributed to melatonin’s role in enhancing insulin biosynthesis and its potent antioxidant properties. Specifically, melatonin has been reported to stimulate insulin secretion, promote pancreatic β-cell regeneration, and protect β-cells from glucotoxicity through oxidative stress mitigation (31). Another experimental study (32) found that melatonin reduced HbA1c levels by decreasing aldehyde and ketone protein derivatives, further supporting its antioxidative mechanism. Clinical evidence also aligns with these findings. Al-Mahbashi et al (33) reported that melatonin supplementation in diabetic patients lowered plasma and erythrocyte malondialdehyde (MDA) levels while increasing glutathione (GSH) concentrations, suggesting that its effect on HbA1c may be mediated via antioxidant pathways. In addition, a randomized crossover study investigating melatonin’s effect on HbA1c was excluded from the current meta-analysis due to methodological limitations. The study failed to provide stage-specific intergroup comparisons and only reported pre- and post-intervention outcomes for the overall population. Moreover, the lack of reporting on potential crossover effects and inadequate control of the washout period further reduced its methodological rigor and limited its contribution to the pooled evidence. While the study suggested a reduction in HbA1c following melatonin intervention, its findings require cautious interpretation. In conclusion, although this meta-analysis suggests that melatonin may exert a beneficial effect on HbA1c in patients with T2DM, the current body of evidence remains limited by methodological constraints, small sample sizes, and variable study quality. Therefore, additional well-designed, large-scale RCTs are warranted to validate these findings and clarify the underlying mechanisms.

There remains considerable controversy in the current literature regarding the effects of melatonin on FPG in individuals with T2DM. This inconsistency may be attributed to melatonin’s bidirectional regulatory effects on pancreatic β-cell function. On one hand, melatonin may suppress insulin secretion through activation of Gi protein-coupled MT1 and MT2 receptors, which inhibits cyclic adenosine monophosphate (cAMP) synthesis and downstream protein kinase A (PKA) activation. This inhibitory pathway may partially explain the elevation in FPG observed in some clinical settings. On the other hand, melatonin has also been shown to stimulate insulin release by activating phospholipase C and inositol triphosphate (IP_3_) signaling via the Gq-coupled MT2 receptor, particularly in individuals with preserved β-cell function (34).These mechanistic differences suggest that the glycemic response to melatonin may be influenced by patient-specific factors such as disease duration, residual β-cell function, and the melatonin administration regimen (including dosage and timing). Moreover, interindividual variability in melatonin metabolism may further contribute to heterogeneous clinical outcomes. Genetic evidence supports this variability; for example, the MTNR1B gene polymorphism rs10830963 has been associated with an increased risk of T2DM. Carriers of the G allele tend to exhibit elevated FPG and HbA1c levels, along with impaired β-cell function (35). Although preliminary findings suggest that melatonin may confer metabolic benefits in selected subpopulations, many existing trials are limited by short intervention durations (≤12 weeks), lack of stratification by key biomarkers (e.g., HOMA-β, MTNR1B genotype), and inadequate subgroup analyses. Future research should focus on well-designed, long-term RCTs that incorporate precision medicine approaches to evaluate the efficacy and safety of melatonin in clearly defined T2DM phenotypes.

Insulin sensitivity was found to decrease by approximately 12% following melatonin treatment compared to placebo (25). This reduction may be attributable to elevated daytime melatonin concentrations resulting from exogenous supplementation. Supporting this, another study demonstrated that acute melatonin administration impaired glucose tolerance both in the morning and at night, indicating that melatonin may reduce insulin sensitivity under specific physiological conditions.In the context of T2DM and related metabolic disorders, the presence of proinflammatory cytokines, oxidative stress, and abnormal protein processing activates inflammatory signaling cascades. These processes contribute to the development of low-grade chronic inflammation in peripheral tissues, which is closely associated with insulin resistance and reduced insulin sensitivity (36).

The use of higher melatonin doses during the night may lead to residual hormone levels extending into the daytime (37). When circulating melatonin remains elevated during daylight hours or meal times, it may interfere with normal glucose metabolism, resulting in increased glycemic variability.Genetic factors also appear to modulate individual responses to melatonin. In particular, the MTNR1B gene polymorphism rs10830963 has been shown to influence melatonin sensitivity. Individuals with the G allele variant exhibit heightened sensitivity to melatonin, impaired insulin secretion, and a greater risk of developing T2DM (38). These findings highlight the importance of considering both pharmacokinetic profiles and genetic background when evaluating melatonin’s role in glycemic control.

## 5 Conclusion

Melatonin supplementation appears to modestly reduce HbA1c levels in patients with T2DM, indicating potential benefits for long-term glycemic control. However, it shows no significant effect on fasting glucose, suggesting limited short-term metabolic impact. Given the lack of long-term safety data, future research should focus on extended, high-quality trials and explore underlying mechanisms to clarify its therapeutic role.

## Data Availability

The original contributions presented in the study are included in the article/**Supplementary Material**. Further inquiries can be directed to the corresponding authors.
