# Supplementary material for: The effect of melatonin supplementation on glycemic control in patients with type 2 diabetes
Source: Front Endocrinol (Lausanne). 2025 Jul 8;16:1572613. doi: 10.3389/fendo.2025.1572613 (PMC12279524; doi:10.3389/fendo.2025.1572613)
Supplement: Supplementary file 1 [file Table1.pdf]

PRISMA 2020 Checklist

Section and Topic	Item #	Checklist item	Location where item is reported
**TITLE**			
Title	1	The Effect of Melatonin Supplementation on Glycemic Control in Patients with Type 2 Diabetes	title
**ABSTRACT**			
Abstract	2	BACKGROUND AND PURPOSE: Melatonin supplementation has shown potential benefits in the management of diabetes in clinical trials; however, prior meta-analyses have not specifically focused on individuals with type 2 diabetes mellitus (T2DM). This study investigates the efficacy of melatonin supplementation in improving glycemic control among patients with T2DM by systematically reviewing and analyzing data from randomized controlled trials (RCTs).<br>METHODS: A comprehensive literature search was conducted in PubMed, Cochrane Library, Scopus, Web of Science, and Embase from their inception to September 2024. RCTs evaluating the effects of melatonin supplementation in adults diagnosed with T2DM were included. The methodological quality of the studies was assessed using the Cochrane Risk of Bias Tool. Data were synthesized and analyzed using RevMan version 5.3.<br>RESULTS: A total of nine RCTs were included in the meta-analysis (n=9). These studies collectively involved 427 participants. Melatonin supplementation was associated with a statistically significant reduction in glycated hemoglobin (HbA1c) levels compared to placebo [mean difference [MD]: -0.65; 95% CI: -1.28, 0.02; P = 0.04], However, no significant effect was observed on fasting plasma glucose (FPG) levels [mean difference: -6.40; 95% CI: -15.79, 2.99; P = 0.18].<br>CONCLUSION: This meta-analysis suggests that melatonin supplementation significantly reduces HbA1c levels in patients with type 2 diabetes mellitus compared to placebo, indicating potential benefits for long-term glycemic control. However, no significant effect was observed on FPG levels.	abstract
**INTRODUCTION**			
Rationale	3	Type 2 diabetes mellitus (T2DM) is a chronic metabolic disorder characterized by insulin resistance and progressive β-cell dysfunction, leading to sustained hyperglycemia and multiple systemic complications. Over the past decades, the global prevalence of T2DM has risen sharply, reflecting both demographic transitions and lifestyle changes. In 2000, diabetes affected approximately 2.8% of the global population; by 2030, this figure is projected to increase to 4.4% (1). As of 2017, diabetes ranked as the ninth leading cause of mortality, with an incidence of 6,059 cases per 100,000 population and more than one million diabetes-related deaths reported annually(2). Notably, the burden of T2DM is shifting toward developing countries, where over 75% of individuals with diabetes are expected to reside by 2025(3).<br>The clinical burden of T2DM extends far beyond hyperglycemia. Long-term complications such as cardiovascular disease, nephropathy, retinopathy, and neuropathy significantly compromise quality of life and increase the risk of disability and premature death(4). Individuals with T2DM face a threefold higher risk of developing cardiovascular disease compared to those without diabetes(5). While pharmacological treatments—particularly oral hypoglycemic agents —remain the mainstay of T2DM management, their use is often associated with reduced efficacy over time, adverse effects (e.g., gastrointestinal discomfort), and, in some cases, toxicity(6). These limitations have prompted growing interest in adjunctive or alternative therapies aimed at improving glycemic control and mitigating complications with fewer side effects.<br>Melatonin, an indoleamine primarily secreted by the pineal gland, plays a central role in the regulation of circadian rhythms and the sleep-wake cycle, and is widely recognized for its potent antioxidant and anti-inflammatory properties(7). Beyond its chronobiological functions, melatonin contributes to cellular protection by scavenging free radicals, attenuating oxidative stress, and modulating the activity of antioxidant enzymes(8,9). Recent research has expanded the potential therapeutic scope of melatonin, suggesting its involvement in glucose metabolism and insulin regulation. Mechanistic studies indicate that melatonin may influence the pathophysiology of T2DM via multiple intracellular signaling pathways, including cyclic adenosine monophosphate (cAMP), cyclic guanosine monophosphate (cGMP), inositol trisphosphate (IP3), and transcription factor-regulated cascades(10). These findings have prompted interest in melatonin as a possible adjunctive intervention in T2DM management.However, the role of melatonin in glucose homeostasis remains controversial. Some evidence suggests that elevated melatonin levels may impair insulin secretion, particularly among individuals with specific genetic polymorphisms. For example, a study from Lund University demonstrated that increased melatonin concentrations were associated with reduced insulin secretion, with this effect being more pronounced in carriers of certain MTNR1B gene variants(11).Although previous meta-analyses(12,13) have examined the association between melatonin supplementation and metabolic parameters in diabetic populations, these studies present two notable limitations. First, they did not differentiate between type 1 diabetes mellitus and T2DM, instead pooling data from both conditions. This methodological approach overlooks critical differences in pathophysiological mechanisms and melatonin responsiveness between the two disease types(14). For instance, insulin resistance—characteristic of T2DM—may alter melatonin receptor signaling pathways, potentially leading to differential therapeutic outcomes(15). Second, several randomized controlled trials (RCTs) published in recent years have specifically investigated the effects of melatonin supplementation in individuals with	Introduction

PRISMA 2020 Checklist

Section and Topic	Item #	Checklist item	Location where item is reported
		T2DM. However, these studies have not yet been comprehensively synthesized in a dedicated meta-analysis. Given the emergence of these new data and the need to clarify melatonin's role in the context of T2DM specifically, an updated and focused meta-analysis is warranted to provide a more accurate and clinically relevant evaluation.	
Objectives	4	This study systematically identified, reviewed, and synthesized RCTs assessing the efficacy of melatonin supplementation in the management of T2DM. Through a comprehensive meta-analysis, it aims to provide an updated and evidence-based evaluation of melatonin's therapeutic potential, thereby informing clinical decision-making and guiding future research directions.	Introduction
**METHODS**			
Eligibility criteria	5	Inclusion Criteria: (1)This study examined the effects of melatonin supplementation on patients with T2DM using a RCT design, published in any language; (2) The study involved patients with T2DM of any gender, age, or nationality; (3) The intervention involved melatonin supplementation, either alone or in combination with other agents; (4) The primary outcomes included glycemic parameters, such as glycated hemoglobin (HbA1c), fasting plasma glucose (FPG), and insulin resistance.<br>Exclusion criteria: (1) Studies involving patients without T2DM or those receiving other interventions unrelated to melatonin supplementation; (2) Research for which there was no complete text available or where the outcome measures were not complete; (3) Studies that were not RCTs, including systematic reviews, mechanistic studies, conference abstracts, and animal studies.	Inclusion and Exclusion Criteria
Information sources	6	A comprehensive literature search was performed across five major electronic databases: PubMed, Cochrane Library, Scopus, Web of Science, and Embase, covering all available records from database inception to September 2024.	Literature Search Methods
Search strategy	7	Pubmend：<br>Search: ((("Diabetes Mellitus, Type 2"[Mesh]) OR (((((((((((((((((((((((((((((((Diabetes Mellitus, Adult-Onset[Title/Abstract]) OR (Adult-Onset Diabetes Mellitus[Title/Abstract])) OR (Diabetes Mellitus, Adult Onset[Title/Abstract])) OR (Diabetes Mellitus, Ketosis-Resistant[Title/Abstract])) OR (Diabetes Mellitus, Ketosis Resistant[Title/Abstract])) OR (Ketosis-Resistant Diabetes Mellitus[Title/Abstract])) OR (Diabetes Mellitus, Non Insulin Dependent[Title/Abstract])) OR (Diabetes Mellitus, Non-Insulin-Dependent[Title/Abstract])) OR (Non-Insulin-Dependent Diabetes Mellitus[Title/Abstract])) OR (Diabetes Mellitus, Stable[Title/Abstract])) OR (Stable Diabetes Mellitus[Title/Abstract])) OR (Diabetes Mellitus, Type II[Title/Abstract])) OR (NIDDM[Title/Abstract])) OR (Diabetes Mellitus, Noninsulin Dependent[Title/Abstract])) OR (Diabetes Mellitus, Maturity-Onset[Title/Abstract])) OR (Diabetes Mellitus, Maturity Onset[Title/Abstract])) OR (Maturity-Onset Diabetes Mellitus[Title/Abstract])) OR (Maturity Onset Diabetes Mellitus[Title/Abstract])) OR (MODY[Title/Abstract])) OR (Diabetes Mellitus, Slow-Onset[Title/Abstract])) OR (Diabetes Mellitus, Slow Onset[Title/Abstract])) OR (Slow-Onset Diabetes Mellitus[Title/Abstract])) OR (Type 2 Diabetes Mellitus[Title/Abstract])) OR (Noninsulin-Dependent Diabetes Mellitus[Title/Abstract])) OR (Noninsulin Dependent Diabetes Mellitus[Title/Abstract])) OR (Maturity-Onset Diabetes[Title/Abstract])) OR (Diabetes, Maturity-Onset[Title/Abstract])) OR (Maturity Onset Diabetes[Title/Abstract])) OR (Maturity Onset Diabetes[Title/Abstract])) OR (Diabetes, Type 2[Title/Abstract])) OR (Diabetes Mellitus, Noninsulin-Dependent[Title/Abstract])))) AND ("Melatonin"[Mesh])) AND (randomized controlled trial[Publication Type] OR randomized[Title/Abstract] OR placebo[Title/Abstract])<br>Cochrane<br>("Diabetes Mellitus, Adult-Onset"):ti,ab,kw OR ("Diabetes Mellitus, Adult-Onset"):ti,ab,kw OR ("Adult-Onset Diabetes Mellitus"):ti,ab,kw OR ("Diabetes Mellitus, Adult Onset"):ti,ab,kw OR ("Diabetes Mellitus, Ketosis-Resistant"):ti,ab,kw OR ("Ketosis-Resistant Diabetes Mellitus"):ti,ab,kw OR ("Diabetes Mellitus, Non Insulin Dependent"):ti,ab,kw OR ("Diabetes Mellitus, Non-Insulin-Dependent"):ti,ab,kw OR ("Non-Insulin-Dependent Diabetes Mellitus"):ti,ab,kw OR ("Diabetes Mellitus, Stable"):ti,ab,kw OR ("Stable Diabetes Mellitus"):ti,ab,kw OR ("Diabetes Mellitus, Type II"):ti,ab,kw OR ("NIDDM"):ti,ab,kw OR ("Diabetes Mellitus, Noninsulin Dependent"):ti,ab,kw OR ("Diabetes Mellitus, Maturity-Onset"):ti,ab,kw OR ("Diabetes Mellitus, Slow Onset"):ti,ab,kw OR ("Slow-Onset Diabetes Mellitus"):ti,ab,kw OR ("Type 2 Diabetes Mellitus"):ti,ab,kw OR ("Noninsulin-Dependent Diabetes Mellitus"):ti,ab,kw OR ("Noninsulin Dependent Diabetes Mellitus"):ti,ab,kw OR ("Maturity-Onset Diabetes"):ti,ab,kw OR ("Diabetes, Maturity-Onset"):ti,ab,kw OR ("Maturity Onset Diabetes"):ti,ab,kw OR ("Type 2 Diabetes"):ti,ab,kw OR ("Diabetes, Type 2"):ti,ab,kw OR ("Diabetes Mellitus, Noninsulin-Dependent"):ti,ab,kw<br>Embase:<br>No. Query Results Results Date	Supplementary Materials - Search Strategies

Section and Topic	Item #	Checklist item	Location where item is reported
		#7. #5 AND #6 51 1 Sep 2024<br>#6. 'randomized controlled trial':ab,ti OR 'randomized':ab,ti OR 'placebo':ab,ti 1,211,970 1 Sep 2024<br>#5. #3 AND #4 703 1 Sep 2024<br>#4. 'melatonin'/exp 45,954 1 Sep 2024<br>#3. #1 OR #2 442,434 1 Sep 2024<br>#2. 'adult onset diabetes':ab,ti OR 'adult onset diabetes mellitus':ab,ti OR 'diabetes mellitus type 2':ab,ti OR 'diabetes mellitus type ii':ab,ti OR 'diabetes mellitus, maturity onset':ab,ti OR 'diabetes mellitus, non insulin dependent':ab,ti OR 'diabetes mellitus, non-insulin-dependent':ab,ti OR 'diabetes mellitus, type 2':ab,ti OR 'diabetes mellitus, type ii':ab,ti OR 'diabetes type 2':ab,ti OR 'diabetes type ii':ab,ti OR 'diabetes, adult onset':ab,ti OR 'dm 2':ab,ti OR 'insulin independent diabetes':ab,ti OR 'insulin independent diabetes mellitus':ab,ti OR 'ketosis resistant diabetes mellitus':ab,ti OR 'maturity onset diabetes':ab,ti OR 'maturity onset diabetes mellitus':ab,ti OR 'niddm':ab,ti OR 'niddm (non insulin dependent diabetes mellitus)':ab,ti OR 'non insulin dependent (type 2) diabetes mellitus':ab,ti OR 'non insulin dependent diabetes':ab,ti OR 'non-insulin-dependent diabetes mellitus':ab,ti OR 'noninsulin dependent (type 2) diabetes mellitus':ab,ti OR 'noninsulin dependent diabetes':ab,ti OR 'noninsulin dependent diabetes mellitus':ab,ti OR 't2dm':ab,ti OR 'tiidm':ab,ti OR 'type 2 (insulin independent) diabetes':ab,ti OR 'type 2 diabetes':ab,ti OR 'type 2 diabetes mellitus':ab,ti OR 'type ii diabetes':ab,ti OR 'type ii diabetes mellitus':ab,ti OR 'non insulin dependent diabetes mellitus' 426,731 1 Sep 2024<br>#1. 'non insulin dependent diabetes mellitus'/exp 383,232 1 Sep 2024<br>Web of Science:<br>TS=(“Diabetes Mellitus, Adult-Onset” OR “Adult-Onset Diabetes Mellitus” OR “Diabetes Mellitus, Adult Onset” OR “Diabetes Mellitus, Ketosis-Resistant” OR “Diabetes Mellitus, Ketosis Resistant” OR “Ketosis-Resistant Diabetes Mellitus” OR “Diabetes Mellitus, Non Insulin Dependent” OR “Diabetes Mellitus,	

PRISMA 2020 Checklist

Section and Topic	Item #	Checklist item	Location where item is reported
		Non-Insulin-Dependent" OR "Non-Insulin-Dependent Diabetes Mellitus" OR "Diabetes Mellitus, Stable" OR "Stable Diabetes Mellitus" OR "Diabetes Mellitus, Type II"OR"NIDDM" OR "Diabetes Mellitus, Noninsulin Dependent" OR "Diabetes Mellitus, Maturity-Onset" OR "Diabetes Mellitus, Maturity Onset" OR "Maturity-Onset Diabetes Mellitus" OR "Maturity Onset Diabetes Mellitus" OR "MODY" OR "Diabetes Mellitus, Slow-Onset" OR "Diabetes Mellitus, Slow Onset" OR "Slow-Onset Diabetes Mellitus" OR "Type 2 Diabetes Mellitus" OR "Noninsulin-Dependent Diabetes Mellitus" OR "Noninsulin Dependent Diabetes Mellitus" OR "Maturity-Onset Diabetes" OR "Diabetes, Maturity-Onset" OR "Maturity Onset Diabetes" OR "Type 2 Diabetes" OR "Diabetes, Type 2" OR "Diabetes Mellitus, Noninsulin-Dependent")<br>TS=(randomized controlled trial OR randomized OR placebo)	
Selection process	8	Two independent reviewers conducted the initial screening of retrieved records by evaluating titles and abstracts against the predefined inclusion and exclusion criteria. Full-text articles were subsequently assessed to determine final eligibility. Any discrepancies between the two reviewers were resolved through discussion, and if necessary, by consulting a third reviewer to reach consensus. EndNote X9 software was employed for reference management.	Literature Screening and Data Extraction
Data collection process	9	Data extraction was carried out using a standardized Microsoft Excel spreadsheet, which was designed to collate retrieved studies, remove duplicates and screen records. This process was performed independently by both reviewers to ensure accuracy and consistency.	Literature Screening and Data Extraction
Data items	10a	Article title, author, year, research design, total sample, age, gender, course of disease, intervention measures, outcome indicators were extracted	Supplementary Material - Data extraction
	10b	Participants were all patients with type 2 diabetes and received either melatonin supplementation or melatonin supplementation plus other interventions.This study was supported by the National Natural Science Foundation of China (81072960/H2902), Natural Science Foundation of Shandong Province(ZR2020MH391), Projects of medical and health technology development program in Shandong province(202104070458)	1.Inclusion and Exclusion Criteria<br>2.Funding
Study risk of bias assessment	11	The methodological quality of the included RCTs was assessed using Review Manager (RevMan) version 5.3, following the guidelines outlined in the Cochrane Handbook for Systematic Reviews of Interventions (version 5.1).	Quality evaluation
Effect measures	12	Effect sizes for continuous variables were expressed as mean differences (MDs) with 95% confidence intervals (CIs).	Statistical analysis
Synthesis methods	13a	Tabulating the study intervention characteristics and the combined effect size was calculated according to the same outcome.	Supplementary Material - Data extraction
	13b	For all included studies, continuous outcomes such as HbA1c and FPG were reported as mean($\bar{X}$) ± standard deviation (SD) values before and after the intervention. When outcomes were presented as standard errors of the mean (SEM), SDs were derived using the formula: SD = SEM × $\sqrt{n}$, where n denotes the sample size of the corresponding group.	Statistical analysis
	13c	The pooled estimate indicated melatonin supplementation showed a beneficial effect on HbA1c compared to placebo [MD: -0.65; 95% CI: -1.28, -0.02; P=0.04]<br>The pooled analysis showed that melatonin supplementation did not lead to a statistically significant reduction in FPG levels compared to placebo [MD = -6.40; 95% CI: -15.79 , 2.99; P = 0.18]	Meta-analysis
	13d	Inter-study heterogeneity was assessed using Cochrane's Q test and the I^2 statistic(18,19). A fixed-effects model was applied when heterogeneity was low ($I^2 \leq$ 50% and P > 0.10), whereas a random-effects model was used in cases of substantial heterogeneity (I^2 > 50% and/or P < 0.10).	Statistical analysis
	13e	There was heterogeneity in the pooled results of HbA1c, and subgroup analysis was performed according to the dose (6mg,10mg). Because there was only one article on 10mg, the subgroup analysis was removed, and the heterogeneity still existed.	Supplementary Material - Data extraction

Section and Topic	Item #	Checklist item	Location where item is reported
	13f	A P > 0.05 was considered indicative of no significant publication bias. Sensitivity analyses were also conducted in Stata to assess the robustness of the pooled results.	Statistical analysis
Reporting bias assessment	14	No evidence of reporting bias was identified.	Quality assessment of the included studies
Certainty assessment	15	Effect sizes for continuous variables were expressed as mean differences (MDs) with 95% confidence intervals (CIs).	Statistical analysis
**RESULTS**			
Study selection	16a	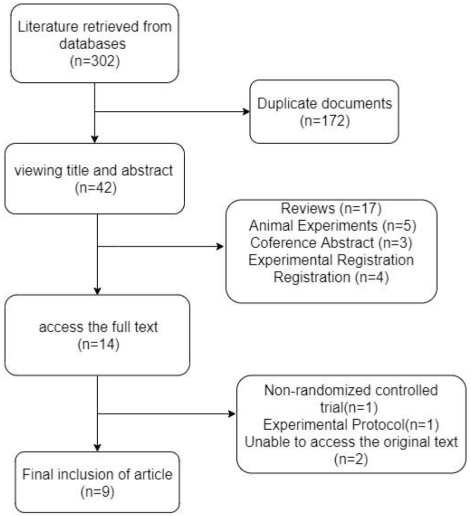	Fig 1. Flowchart of the Screening of Literature
	16b	“Common type 2 diabetes risk variant in MTNR1B worsens the deleterious effect of melatonin on glucose tolerance in humans” is not an RCT. “Effects of Melatonin on Glycemic Variability in Type 2 Diabetes Mellitus: Protocol for a Crossover, Double-Blind, Placebo-Controlled Trial”is the clinical trial protocol.	Supplementary Material - Data extraction

PRISMA 2020 Checklist

Section and Topic	Item #	Checklist item	Location where item is reported
Study characteristics	17	(table below)	Table 1.Detailed description of the basic characteristics and main results of 9 RCTS included

First Author/ Year	Study Design	Sample	Age	Gender (M/F)	Intervention Duration	Intervention	Main Results
Diana-Maria Anton(20) (2021)	Randomized Double- blind	54 patients with T2DM with symptoms of periodontal disease	T:53.24±3.4 C:52.21±3.1	T:60%/40%C: 56%/44%	8 weeks	T:melatonin(6mg) C:placebo(6mg)	①
Bazyar, Hadi(21) (2022)	Randomized Double- blind	50 patients with type 2 diabetes with symptoms of periodontal disease	T:53.72±6.68 C:51.45±0.03	T:46.75%/53. 3% C:53.3%/46.7 %	8 weeks	T:melatonin(6mg) C:placebo(6mg)	①
Amir Farrokhian(22) (2019)	Randomized Triple-blind	70 patients with type 2 diabetes	T:57.74±8.57 C:57.61±9.11	T:17/17 C:20/16	8 weeks	T:melatonin(6mg) C:placebo(6mg)	①②
Doron Garfinkel(23) (2011)	Randomized Double- blind Crossover	36 patients with type 2 diabetes mellitus with insomnia	63±8	11/25	7weeks+20week s	melatonin(2mg) - elution - placebo(2mg)/placebo(2 mg)- elution -melatonin(2mg) melatonin(2mg)	①
Saad A Hussain(24) (2006)	Randomized Double- blind	46 patients with type 2 diabetes	49.1±6.0	A: 8/7 B: 11/7 C: 7/6	12 weeks	A:placebo + metformin B:melatonin(10mg) + zinc(50mg) + metformin C:melatonin(10mg) + zinc(50mg)	①②
Esben S. Lauritzen(25) (2022)	Randomized Double- blind Crossover	17 patients with type 2 diabetes	41-70	male patients	12 weeks	melatonin(10mg) - elution - placebo(10mg)	③
Wagner Martorina(26) (2023)	Randomized Double- blind Crossover	30 patients with type 2 diabetes	A:62,27±8,20 B:61,87±10,95	A:6/9 B:8/7	3 weeks	A:melatonin(3mg) - elution - placebo(3mg) B:placebo(3mg)- elution -melatonin(3mg)	④
Fariba Raygan(27) (2019)	Randomized Double- blind	60 patients with type 2 diabetes mellitus coronary heart disease	T:67.7±11.4 C:65.3±10.1	T:16/14 C:17/13	12 weeks	T:melatonin(10mg) C:placebo(10mg)	②⑤
Mohammad Reza Rezvanfar(28) (2016)	Randomized Double- blind	64 patients with type 2 diabetes	52±8	23.5%/76.5%	24 weeks	placebo(6mg)- elution -melatonin(6mg)	①②

①glycated hemoglobin (HbA1c) ②fasting plasma glucose (FPG) ③ insulin sensitivity ④glycemic Variability ⑤quantitative insulin sensitivity check index

<table>
<tr><th>Section and Topic</th><th>Item #</th><th>Checklist item</th><th>Location where item is reported</th></tr>
<tr><td>Risk of bias in studies</td><td>18</td><td>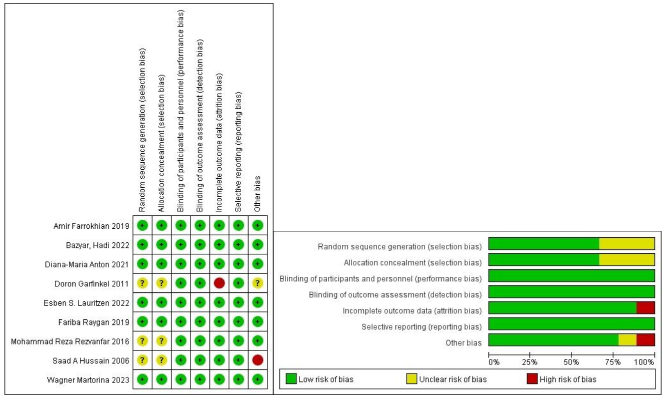
</td><td>Fig5.Summary of the risk of bias in the included studies
Fig6.Graph of the risk of bias in the included studies</td></tr>
<tr><td>Results of individual studies</td><td>19</td><td>Forest plots of Melatonin supplementation's effects on HbA1C
<table>
<tr><th></th><th>Mulatonin</th><th></th><th></th><th>Placebo</th><th></th><th></th><th></th><th>Mean Difference</th></tr>
<tr><th>Study or Subgroup</th><th>Mean</th><th>SD</th><th>Total</th><th>Mean</th><th>SD</th><th>Total</th><th>Weight</th><th>IV, Random, 95% CI</th></tr>
<tr><td>Amir Farrokhian 2019</td><td>7.32</td><td>0.95</td><td>34</td><td>7.08</td><td>1.07</td><td>36</td><td>21.3%</td><td>0.24 [-0.23, 0.71]</td></tr>
<tr><td>Bazyar, Hadi 2022</td><td>7.67</td><td>1.19</td><td>22</td><td>8.03</td><td>1.03</td><td>22</td><td>19.1%</td><td>-0.36 [-1.02, 0.30]</td></tr>
<tr><td>Diana-Maria Anton 2021</td><td>6.3781</td><td>0.31</td><td>25</td><td>7.5823</td><td>0.57</td><td>25</td><td>23.3%</td><td>-1.20 [-1.46, -0.95]</td></tr>
<tr><td>Mohammad Reza Rezvanfar 2016</td><td>7.16</td><td>0.88</td><td>64</td><td>7.5</td><td>0.72</td><td>64</td><td>23.1%</td><td>-0.34 [-0.62, -0.06]</td></tr>
<tr><td>Saad A Hussain 2006</td><td>5.71</td><td>1.36</td><td>18</td><td>7.8</td><td>1.94</td><td>15</td><td>13.2%</td><td>-2.09 [-3.26, -0.92]</td></tr>
<tr><td>Total (95% CI)</td><td></td><td></td><td>163</td><td></td><td></td><td>162</td><td>100.0%</td><td>-0.65 [-1.28, -0.02]</td></tr>
</table>
Heterogeneity: Tau² = 0.43; Chi² = 43.08, df = 4 (P < 0.00001); I² = 91%
Test for overall effect: Z = 2.03 (P = 0.04)
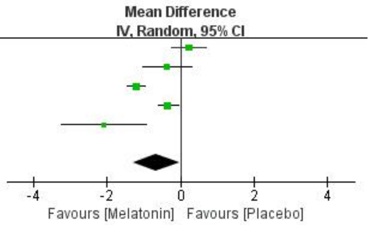

Forest plots of Melatonin supplementation's effects on FPG
<table>
<tr><th></th><th>Melatonin</th><th></th><th></th><th>Placebo</th><th></th><th></th><th></th><th>Mean Difference</th></tr>
<tr><th>Study or Subgroup</th><th>Mean</th><th>SD</th><th>Total</th><th>Mean</th><th>SD</th><th>Total</th><th>Weight</th><th>IV, Fixed, 95% CI</th></tr>
<tr><td>Amir Farrokhian 2019</td><td>138.12</td><td>31.05</td><td>34</td><td>137.72</td><td>28.65</td><td>36</td><td>44.9%</td><td>0.40 [-13.62, 14.42]</td></tr>
<tr><td>Fariba Raygan 2019</td><td>141.5</td><td>49.2</td><td>30</td><td>151</td><td>55</td><td>30</td><td>12.6%</td><td>-9.50 [-35.91, 16.91]</td></tr>
<tr><td>Mohammad Reza Rezvanfar 2016</td><td>146.8</td><td>44.8</td><td>64</td><td>157.5</td><td>44</td><td>64</td><td>37.2%</td><td>-10.70 [-26.08, 4.68]</td></tr>
<tr><td>Saad A Hussain 2006</td><td>140.94</td><td>41.22</td><td>18</td><td>167.4</td><td>71.64</td><td>15</td><td>5.3%</td><td>-26.46 [-67.41, 14.49]</td></tr>
<tr><td>Total (95% CI)</td><td></td><td></td><td>146</td><td></td><td></td><td>145</td><td>100.0%</td><td>-6.40 [-15.79, 2.99]</td></tr>
</table>
Heterogeneity: Chi² = 2.18, df = 3 (P = 0.54); I² = 0%
Test for overall effect: Z = 1.34 (P = 0.18)
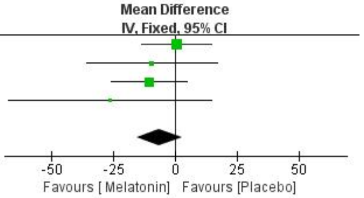
</td><td>Fig2. Forest plots of Melatonin supplementation's effects on HbA1C
Fig4. Forest plots of Melatonin supplementation's effects on FPG</td></tr>
</table>

PRISMA 2020 Checklist

Section and Topic	Item #	Checklist item	Location where item is reported
Results of syntheses	20a	(see table below)	Supplementary Material - Data extraction

First Author/ Year	Intervention	Main Results	randomized sequence generation	allocation concealment	blinding of participants and trial personnel	blinding of outcome assessors	completeness of outcome data	selective outcome reporting	other biases
Amir Farrokhian[19] (2019)	T:melatonin(6mg) C:placebo(6mg)	HbA1c FPG HOMA-IR	low risk	low risk	low risk	low risk	low risk	low risk	low risk
Bazyar, Hadi[18] (2022)	T:melatonin(6mg) C:placebo(6mg)	HbA1c	low risk	low risk	low risk	low risk	low risk	low risk	low risk
Diana-Maria Anton[17] (2021)	T:melatonin(6mg) C:placebo(6mg)	HbA1c	low risk	low risk	low risk	low risk	low risk	low risk	low risk
Mohammad Reza Rezvanfar[25] (2016)	placebo(6mg)-elution -melatonin(6mg)	HbA1c FPG	unclear.	unclear.	low risk	low risk	low risk	low risk	low risk
Saad A Hussain[21] (2006)	A:placebo + metformin B:melatonin(10mg) + zinc(50mg) + metformin C:melatonin(10mg) + zinc(50mg)	HbA1c FPG	unclear.	unclear.	low risk	low risk	low risk	low risk	high risk

PRISMA 2020 Checklist

Section and Topic	Item #	Checklist item	Location where item is reported

First Author/ Year	Intervention	Main Results	randomized sequence generation	allocation concealment	blinding of participants and trial personnel	blinding of outcome assessors	completeness of outcome data	selective outcome reporting	other biases
Amir Farrokhian[19] (2019)	T:melatonin(6mg) C:placebo(6mg)	HbA1c FPG HOMA-IR	low risk	low risk	low risk	low risk	low risk	low risk	low risk
Fariba Raygan[24] (2019)	T:melatonin(10mg) C:placebo(10mg)	FPG HOMA-IR QUICKI	low risk	low risk	low risk	low risk	low risk	low risk	low risk
Mohammad Reza Rezvanfar[25] (2016)	placebo(6mg)-elution -melatonin(6mg)	HbA1c FPG	unclear.	unclear.	low risk	low risk	low risk	low risk	low risk
Saad A Hussain[21] (2006)	A:placebo + metformin B:melatonin(10mg) + zinc(50mg) + metformin C:melatonin(10mg) + zinc(50mg)	HbA1c FPG	unclear.	unclear.	low risk	low risk	low risk	low risk	high risk

Section and Topic	Item #	Checklist item	Location where item is reported
	20b	A fixed-effects model was applied when heterogeneity was low ($I^2 \leq 50\%$ and $P > 0.10$), whereas a random-effects model was used in cases of substantial heterogeneity ($I^2 > 50\%$ and/or $P < 0.10$).<br>HbA1c: Significant heterogeneity was detected among studies ($P < 0.00001$, $I^2 = 91\%$), warranting the use of a random-effects model for meta-analysis. .<br>FPG: Heterogeneity analysis indicated no significant between-study variability ($P = 0.54$; $I^2 = 0\%$), justifying the use of a fixed-effects model.	1.Statistical analysis<br>2.Meta-analysis
	20c	No sources of heterogeneity for Hba1c were identified. There was no heterogeneity in FPG	Meta-analysis
	20d	Sensitivity analysis of HbA1c<br>However, given the proximity of the confidence interval upper limit to the null value and the borderline statistical significance, these	Meta-analysis

Section and Topic	Item #	Checklist item	Location where item is reported
		findings should be interpreted with caution. Sensitivity analysis, conducted via a leave-one-out approach, demonstrated consistent directionality of effect with all pooled MDs remaining negative and 95% CIs not crossing zero—the highest upper bound reaching only -0.08 (Fig 3). Notably, the exclusion of the study by Diana-Maria Anton (2021) resulted in a confidence interval with an upper limit closest to zero, indicating this study had the greatest influence on the pooled estimate. However, its removal did not reverse the direction of the effect. The exclusion of other individual studies yielded minimal changes, implying limited influence on the overall meta-analytic estimate. 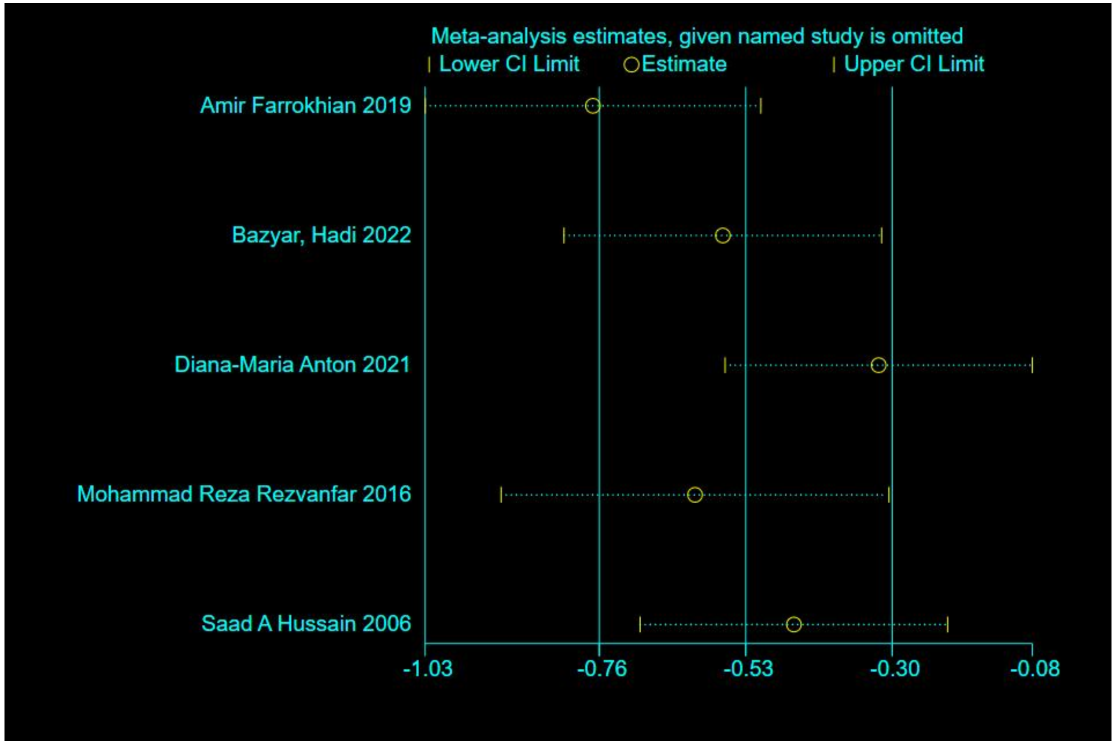	
Reporting biases	21	No evidence of reporting bias was identified.	Quality assessment of the included studies
Certainty of evidence	22	The pooled estimate indicated melatonin supplementation showed a beneficial effect on HbA1c compared to placebo [MD: -0.65; 95% CI: -1.28, -0.02; P=0.04]<br>The pooled analysis showed that melatonin supplementation did not lead to a statistically significant reduction in FPG levels compared to placebo [MD = -6.40; 95% CI: -15.79 , 2.99; P = 0.18]	Meta-analysis
**DISCUSSION**			
Discussion	23a	Preclinical evidence supports the potential of melatonin in modulating glycemic control. An animal study(30) demonstrated that melatonin treatment significantly improved hyperglycemia in T2DM rat models by reducing insulin levels, inhibiting hemoglobin glycation, and ameliorating insulin resistance.	Discussion

Section and Topic	Item #	Checklist item	Location where item is reported
		These effects may be attributed to melatonin's role in enhancing insulin biosynthesis and its potent antioxidant properties. Specifically, melatonin has been reported to stimulate insulin secretion, promote pancreatic β-cell regeneration, and protect β-cells from glucotoxicity through oxidative stress mitigation(31). Another experimental study(32) found that melatonin reduced HbA1c levels by decreasing aldehyde and ketone protein derivatives, further supporting its antioxidative mechanism. Clinical evidence also aligns with these findings. Al-Mahbashi et al(33) reported that melatonin supplementation in diabetic patients lowered plasma and erythrocyte malondialdehyde (MDA) levels while increasing glutathione (GSH) concentrations, suggesting that its effect on HbA1c may be mediated via antioxidant pathways. In addition, a randomized crossover study investigating melatonin's effect on HbA1c was excluded from the current meta-analysis due to methodological limitations. The study failed to provide stage-specific intergroup comparisons and only reported pre- and post-intervention outcomes for the overall population. Moreover, the lack of reporting on potential crossover effects and inadequate control of the washout period further reduced its methodological rigor and limited its contribution to the pooled evidence. While the study suggested a reduction in HbA1c following melatonin intervention, its findings require cautious interpretation. In conclusion, although this meta-analysis suggests that melatonin may exert a beneficial effect on HbA1c in patients with T2DM, the current body of evidence remains limited by methodological constraints, small sample sizes, and variable study quality. Therefore, additional well-designed, large-scale RCTs are warranted to validate these findings and clarify the underlying mechanisms.<br>There remains considerable controversy in the current literature regarding the effects of melatonin on FPG in individuals with T2DM. This inconsistency may be attributed to melatonin's bidirectional regulatory effects on pancreatic β-cell function. On one hand, melatonin may suppress insulin secretion through activation of Gi protein-coupled MT1 and MT2 receptors, which inhibits cyclic adenosine monophosphate (cAMP) synthesis and downstream protein kinase A (PKA) activation. This inhibitory pathway may partially explain the elevation in FPG observed in some clinical settings. On the other hand, melatonin has also been shown to stimulate insulin release by activating phospholipase C and inositol triphosphate (IP_3) signaling via the Gq-coupled MT2 receptor, particularly in individuals with preserved β-cell function(34).These mechanistic differences suggest that the glycemic response to melatonin may be influenced by patient-specific factors such as disease duration, residual β-cell function, and the melatonin administration regimen (including dosage and timing). Moreover, interindividual variability in melatonin metabolism may further contribute to heterogeneous clinical outcomes. Genetic evidence supports this variability; for example, the MTNR1B gene polymorphism rs10830963 has been associated with an increased risk of T2DM. Carriers of the G allele tend to exhibit elevated FPG and HbA1c levels, along with impaired β-cell function(35). Although preliminary findings suggest that melatonin may confer metabolic benefits in selected subpopulations, many existing trials are limited by short intervention durations (≤12 weeks), lack of stratification by key biomarkers (e.g., HOMA-β, MTNR1B genotype), and inadequate subgroup analyses. Future research should focus on well-designed, long-term RCTs that incorporate precision medicine approaches to evaluate the efficacy and safety of melatonin in clearly defined T2DM phenotypes.	
	23b	The limitations of this paper are that few articles were included, and no meta-analysis or subgroup analysis could be performed for some of the results	Supplementary Material - Data extraction
	23c	The full text was not available for some articles, and subgroup analyses were not available for some articles	Supplementary Material - Data extraction
	23d	Melatonin supplementation appears to modestly reduce HbA1c levels in patients with T2DM, indicating potential benefits for long-term glycemic control. However, it shows no significant effect on fasting glucose, suggesting limited short-term metabolic impact. Given the lack of long-term safety data, future research should focus on extended, high-quality trials and explore underlying mechanisms to clarify its therapeutic role.	Conclusion
**OTHER INFORMATION**			
Registration and protocol	24a	I have applied for registration, and the registered person is Sun Huiqi. Registered platform PROSPERO, registration number CRD42024629557	https://www.crd.york.ac.uk/PROSPERO/
	24b	Check out this website for https://www.crd.york.ac.uk/PROSPERO/	https://www.crd.york.ac.uk/PROSPERO/
	24c	Not modified during the registration process.	https://www.crd.york.ac.uk/PROSP

PRISMA 2020 Checklist

Section and Topic	Item #	Checklist item	Location where item is reported
			ERO/
Support	25	This study was supported by the National Natural Science Foundation of China (81072960/H2902), Natural Science Foundation of Shandong Province(ZR2020MH391), Projects of medical and health technology development program in Shandong province(202104070458).	Funding
Competing interests	26	The authors declare that the research was conducted in the absence of any commercial or financial relationships that could be construed as a potential conflict of interest.	Conflict of Interest
Availability of data, code and other materials	27	Report which of the following are publicly available and where they can be found: template data collection forms; data extracted from included studies; data used for all analysesare not publicly available. Any other materials used in the review is available by contacting the authors.	Supplementary Material - Data extraction

From: Page MJ, McKenzie JE, Bossuyt PM, Boutron I, Hoffmann TC, Mulrow CD, et al. The PRISMA 2020 statement: an updated guideline for reporting systematic reviews. BMJ 2021;372:n71. doi: 10.1136/bmj.n71. This work is licensed under CC BY 4.0. To view a copy of this license, visit https://creativecommons.org/licenses/by/4.0/
