# Supplementary material for: The effect of melatonin supplementation on glycemic control in patients with type 2 diabetes
Source: Front Endocrinol (Lausanne). 2025 Jul 8;16:1572613. doi: 10.3389/fendo.2025.1572613 (PMC12279524; doi:10.3389/fendo.2025.1572613)
Supplement: Supplementary file 2 [file DataSheet1.docx]

Supplementary Material

# Search strategy

PubMed:

Search: ((("Diabetes Mellitus, Type 2"[Mesh]) OR (((((((((((((((((((((((((((((((Diabetes Mellitus, Adult-Onset[Title/Abstract]) OR (Adult-Onset Diabetes Mellitus[Title/Abstract])) OR (Diabetes Mellitus, Adult Onset[Title/Abstract])) OR (Diabetes Mellitus, Ketosis-Resistant[Title/Abstract])) OR (Diabetes Mellitus, Ketosis Resistant[Title/Abstract])) OR (Ketosis-Resistant Diabetes Mellitus[Title/Abstract])) OR (Diabetes Mellitus, Non Insulin Dependent[Title/Abstract])) OR (Diabetes Mellitus, Non-Insulin-Dependent[Title/Abstract])) OR (Non-Insulin-Dependent Diabetes Mellitus[Title/Abstract])) OR (Diabetes Mellitus, Stable[Title/Abstract])) OR (Stable Diabetes Mellitus[Title/Abstract])) OR (Diabetes Mellitus, Type II[Title/Abstract])) OR (NIDDM[Title/Abstract])) OR (Diabetes Mellitus, Noninsulin Dependent[Title/Abstract])) OR (Diabetes Mellitus, Maturity-Onset[Title/Abstract])) OR (Diabetes Mellitus, Maturity Onset[Title/Abstract])) OR (Maturity-Onset Diabetes Mellitus[Title/Abstract])) OR (Maturity Onset Diabetes Mellitus[Title/Abstract])) OR (MODY[Title/Abstract])) OR (Diabetes Mellitus, Slow-Onset[Title/Abstract])) OR (Diabetes Mellitus, Slow Onset[Title/Abstract])) OR (Slow-Onset Diabetes Mellitus[Title/Abstract])) OR (Type 2 Diabetes Mellitus[Title/Abstract])) OR (Noninsulin-Dependent Diabetes Mellitus[Title/Abstract])) OR (Noninsulin Dependent Diabetes Mellitus[Title/Abstract])) OR (Maturity-Onset Diabetes[Title/Abstract])) OR (Diabetes, Maturity-Onset[Title/Abstract])) OR (Maturity Onset Diabetes[Title/Abstract])) OR (Maturity Onset Diabetes[Title/Abstract])) OR (Diabetes, Type 2[Title/Abstract])) OR (Diabetes Mellitus, Noninsulin-Dependent[Title/Abstract]))) AND ("Melatonin"[Mesh])) AND (randomized controlled trial[Publication Type] OR randomized[Title/Abstract] OR placebo[Title/Abstract])

Cochrane:

(“Diabetes Mellitus, Adult-Onset”):ti,ab,kw OR (“Diabetes Mellitus, Adult-Onset”):ti,ab,kw OR (“Adult-Onset Diabetes Mellitus”):ti,ab,kw OR (“Diabetes Mellitus, Adult Onset”):ti,ab,kw OR (“Diabetes Mellitus, Ketosis-Resistant”):ti,ab,kw OR (“Ketosis-Resistant Diabetes Mellitus”):ti,ab,kw OR (“Diabetes Mellitus, Non Insulin Dependent”):ti,ab,kw OR (“Diabetes Mellitus, Non-Insulin-Dependent”):ti,ab,kw OR (“Non-Insulin-Dependent Diabetes Mellitus”):ti,ab,kw OR (“Diabetes Mellitus, Stable”):ti,ab,kw OR (“Stable Diabetes Mellitus”):ti,ab,kw OR (“Diabetes Mellitus, Type II”):ti,ab,kw OR (“NIDDM”):ti,ab,kw OR (“Diabetes Mellitus, Noninsulin Dependent”):ti,ab,kw OR (“Diabetes Mellitus, Maturity-Onset”):ti,ab,kw OR (“Diabetes Mellitus, Slow Onset”):ti,ab,kw OR (“Slow-Onset Diabetes Mellitus”):ti,ab,kw OR (“Type 2 Diabetes Mellitus”):ti,ab,kw OR (“Noninsulin-Dependent Diabetes Mellitus”):ti,ab,kw OR (“Noninsulin Dependent Diabetes Mellitus”):ti,ab,kw OR (“Maturity-Onset Diabetes”):ti,ab,kw OR (“Diabetes, Maturity-Onset”):ti,ab,kw OR (“Maturity Onset Diabetes”):ti,ab,kw OR (“Type 2 Diabetes”):ti,ab,kw OR (“Diabetes, Type 2”):ti,ab,kw OR (“Diabetes Mellitus, Noninsulin-Dependent”):ti,ab,kw

Embase

No. Query Results Results Date

#7. #5 AND #6 51 1 Sep 2024

#6. 'randomized controlled trial':ab,ti OR 1,211,970 1 Sep 2024

'randomized':ab,ti OR 'placebo':ab,ti

#5. #3 AND #4 703 1 Sep 2024

#4. 'melatonin'/exp 45,954 1 Sep 2024

#3. #1 OR #2 442,434 1 Sep 2024

#2. 'adult onset diabetes':ab,ti OR 'adult onset 426,731 1 Sep 2024

diabetes mellitus':ab,ti OR 'diabetes mellitus

type 2':ab,ti OR 'diabetes mellitus type

ii':ab,ti OR 'diabetes mellitus, maturity

onset':ab,ti OR 'diabetes mellitus, non insulin

dependent':ab,ti OR 'diabetes mellitus,

non-insulin-dependent':ab,ti OR 'diabetes

mellitus, type 2':ab,ti OR 'diabetes mellitus,

type ii':ab,ti OR 'diabetes type 2':ab,ti OR

'diabetes type ii':ab,ti OR 'diabetes, adult

onset':ab,ti OR 'dm 2':ab,ti OR 'insulin

independent diabetes':ab,ti OR 'insulin

independent diabetes mellitus':ab,ti OR 'ketosis

resistant diabetes mellitus':ab,ti OR 'maturity

onset diabetes':ab,ti OR 'maturity onset diabetes

mellitus':ab,ti OR 'niddm':ab,ti OR 'niddm (non

insulin dependent diabetes mellitus)':ab,ti OR

'non insulin dependent (type 2) diabetes

mellitus':ab,ti OR 'non insulin dependent

diabetes':ab,ti OR 'non-insulin-dependent

diabetes mellitus':ab,ti OR 'noninsulin dependent

(type 2) diabetes mellitus':ab,ti OR 'noninsulin

dependent diabetes':ab,ti OR 'noninsulin

dependent diabetes mellitus':ab,ti OR

't2dm':ab,ti OR 'tiidm':ab,ti OR 'type 2 (insulin

independent) diabetes':ab,ti OR 'type 2

diabetes':ab,ti OR 'type 2 diabetes

mellitus':ab,ti OR 'type ii diabetes':ab,ti OR

'type ii diabetes mellitus':ab,ti OR 'non insulin

dependent diabetes mellitus'

#1. 'non insulin dependent diabetes mellitus'/exp 383,232 1 Sep 2024

Web of Science:

TS=(“Diabetes Mellitus, Adult-Onset” OR “Adult-Onset Diabetes Mellitus” OR “Diabetes Mellitus, Adult Onset” OR “Diabetes Mellitus, Ketosis-Resistant” OR “Diabetes Mellitus, Ketosis Resistant” OR “Ketosis-Resistant Diabetes Mellitus” OR “Diabetes Mellitus, Non Insulin Dependent” OR “Diabetes Mellitus, Non-Insulin-Dependent” OR “Non-Insulin-Dependent Diabetes Mellitus” OR “Diabetes Mellitus, Stable” OR “Stable Diabetes Mellitus” OR “Diabetes Mellitus, Type II”OR“NIDDM” OR “Diabetes Mellitus, Noninsulin Dependent” OR “Diabetes Mellitus, Maturity-Onset” OR “Diabetes Mellitus, Maturity Onset” OR “Maturity-Onset Diabetes Mellitus” OR “Maturity Onset Diabetes Mellitus” OR “MODY” OR “Diabetes Mellitus, Slow-Onset” OR “Diabetes Mellitus, Slow Onset” OR “Slow-Onset Diabetes Mellitus” OR “Type 2 Diabetes Mellitus” OR “Noninsulin-Dependent Diabetes Mellitus” OR “Noninsulin Dependent Diabetes Mellitus” OR “Maturity-Onset Diabetes” OR “Diabetes, Maturity-Onset” OR “Maturity Onset Diabetes” OR “Type 2 Diabetes” OR “Diabetes, Type 2” OR “Diabetes Mellitus, Noninsulin-Dependent”)

TS=(randomized controlled trial OR randomized OR placebo)
